# Clausmarin A, Potential Immunosuppressant Revealed by Yeast-Based Assay and Interleukin-2 Production Assay in Jurkat T Cells

**DOI:** 10.1371/journal.pone.0136804

**Published:** 2015-08-27

**Authors:** Pitipreya Suauam, Boon-ek Yingyongnarongkul, Tanapat Palaga, Tokichi Miyakawa, Chulee Yompakdee

**Affiliations:** 1 Department of Microbiology, Faculty of Science, Chulalongkorn University, Phayathai Road, Pathumwan, Bangkok, 10330, Thailand; 2 Department of Chemistry and Center of Excellence for Innovation in Chemistry, Faculty of Science, Ramkhamhaeng University, Ramkhamhaeng Road, Hua Mark, Bangkapi, Bangkok, 10240, Thailand; 3 Department of Molecular Biotechnology, Graduate School of Advanced Sciences of Matter, Hiroshima University, Higashihiroshima, 739–8530, Japan; School of Medicine and Health Sciences, University of North Dakota, UNITED STATES

## Abstract

Small-molecule inhibitors of Ca^2+^-signaling pathways are of medicinal importance, as exemplified by the immunosuppressants FK506 and cyclosporin A. Using a yeast-based assay devised for the specific detection of Ca^2+^-signaling inhibitors, clausmarin A, a previously reported terpenoid coumarin, was identified as an active substance. Here, we investigated the likely mechanism of clausmarin A action in yeast and Jurkat T-cells. In the presence of 100 mM CaCl_2_ in the growth medium of Ca^2+^-sensitive Δ*zds1* strain yeast, clausmarin A exhibited a dose-dependent alleviation of various defects due to hyperactivation of Ca^2+^ signaling, such as growth inhibition, polarized bud growth and G2 phase cell-cycle arrest. Furthermore, clausmarin A inhibited the growth of Δ*mpk1* (lacking the Mpk1 MAP kinase pathway) but not Δ*cnb1* (lacking the calcineurin pathway) strain, suggesting that clausmarin A inhibited the calcineurin pathway as presumed from the synthetic lethality of these pathways. Furthermore, clausmarin A alleviated the serious defects of a strain expressing a constitutively active form of calcineurin. In the human Jurkat T-cell line, clausmarin A exhibited a dose-dependent inhibition of IL-2 production and IL-2 gene transcription, as well as an inhibition of NFAT dephosphorylation. The effects of clausmarin A observed in both yeast and Jurkat cells are basically similar to those of FK506. Our study revealed that clausmarin A is an inhibitor of the calcineurin pathway, and that this is probably mediated via inhibition of calcineurin phosphatase activity. As such, clausmarin A is a potential immunosuppressant.

## Introduction

During the process of T cell activation, the activated T cells express interleukin 2 (IL-2), a cytokine that promotes T cell proliferation by interacting with the IL-2 receptors. Several transcription factors, including nuclear factor of activated T-cells (NFAT), have been identified to bind the IL-2 promoter region [1]. The transcription factor NFAT, which plays an essential role in IL-2 expression, is a complex composed of a cytoplasmic subunit and an inducible nuclear component comprised of AP-1 family members. The N terminus of NFAT regulates nuclear/cytoplasm trafficking in response to changes in intracellular calcium ion (Ca^2+^) concentrations. In resting T cells, NFAT is located in the cytoplasm and is heavily phosphorylated. Upon T cell activation, or treatment of cells with Ca^2+^ ionophore, the Ca^2+^/calmodulin-dependent Ser/Thr phosphatase calcineurin phosphatase is activated and dephosphorylates NFAT, resulting in the nuclear translocation of NFAT [2], [3].

The clinically important immunosuppressive drugs, FK506 (Tacrolimus) and cyclosporine A, act by binding to their specific immunophilins FKBP12 and cyclophilin, respectively [2]. The immunophilin-drug complex then binds to calcineurin and inhibits its phosphatase activity and so prevents the dephosphorylation and nuclear translocation of NFAT [4–6]. Both FK506 and cyclosporin A have been shown to be effective immunosuppressant agents in organ transplantation, by preventing organ graft rejection in the clinic [7], and autoimmune diseases. Despite their effectiveness, the long-term use of these drugs has several undesired side effects, such as nephrotoxicity, diabetogenicity, neurotoxicity and gastrointestinal toxicity [8], hyperkalemia and hyperuricemia, which impose serious problems in immunosuppressive therapy [9]. Accordingly, a number of new compounds have been developed for use as immunosuppressive drugs to treat autoimmune diseases and prevent organ rejection in solid organ transplantation that have the advantage of a relatively low toxicity [3], [9], but there is still a requirement for improvement and new compounds.

In the Ca^2+^-sensitive Δ*zds1* yeast (*Saccharomyces cerevisiae*) strain, Ca^2+^ signaling has been implicated in the inhibition of the G2/M cell-cycle progression [10]. On the basis of this mechanism, a unique drug-screening procedure for inhibitors of Ca^2+^-signaling was developed [11]. Since many small molecule inhibitors act in an evolutionally conserved manner throughout eukaryotes, those drugs revealed by this yeast assay system have a reasonable probability of also being effective on human cells by analogous mechanisms that share a drug target molecule.

Using the Δ*zds1* yeast-based assay to detect Ca^2+^-signaling inhibitors, we could detect clausmarin A, a terpenoid coumarin, as a potential bioactive Ca^2+^ signaling inhibitor (our unpublished data). This compound has previously been reported as a component of *Clausena pentaphylla* (Roxb.) DC. and has a spasmolytic activity, but little was known of its action mechanism [12]. To investigate its mechanism of action in detail, we took advantage of the molecular genetic approaches available in yeast, utilizing relevant mutants and genetically manipulated yeast strains. We further examined the effect of clausmarin A using a human IL-2 producing T-cell line, in which a calcineurin-mediated mechanism plays a key regulatory role in the control of IL-2 production. Through these studies, we showed that clausmarin A inhibited the calcineurin-mediated pathway in both yeast and human cells.

## Materials and Methods

### Yeast Strains and Their Cultivation

The *S*. *cerevisiae* strains used in this study are shown in Table 1. The calcium-sensitive Δ*zds1* mutant of *S*. *cerevisiae* (YNS17) was used as the indicator cell in the drug assay [13]. The cells were refreshed before use by cultivation on a YPD (yeast extract-peptone-dextrose) agar plate at 30°C for 2 d. The YPD medium was supplemented with adenine (400 μg/mL) and uracil (200 μg/mL). For over-expression of *CMP2*ΔC, a constitutively active form of calcineurin catalytic subunit, YPGal (yeast extract-peptone-2% (w/v) galactose 1% (w/v) raffinose) medium was used for cultivation of the YRC1 strain. The strains Δ*cnb1* and Δ*mpk1*were cultivated in YPD medium.

**Table 1:** Yeast strains used in this study.

Yeast strain	Genotype	Source
Δ*zds1* (YNS17)	*MAT*a *leu2 ade2 ura3 his3 can1-1 zds1*::*TRP1 erg3*::HIS3 *pdr1*::*hisG-URA3-hisG pdr3*:: *hisG-URA3-hisG*	[13]
Δ*cnb1*	*MATa trp1 leu2 ade2 ura3 his3 can1-1 cnb1*::*HIS3*	Miyakawa T, Hiroshima University
Δ*mpk1*	*MATa trp1 leu2 ade2 ura3 his3 can1-1 mpk1*::*HIS3*	Miyakawa T, Hiroshima University
YRC1	*MAT*a *GAL-CMP2*Δ*C*::*URA3 zds1*::*TRP1 erg3*::*HIS3 pdr1*::*hisG pdr3*::*hisG*	[13]

### Jurkat Human T Cell Line and its Cultivation

The Jurkat T cell line (Human acute T cell leukemia), ATCC number CRL-2063, was obtained from the Institute of Biotechnology and Genetic Engineering, Chulalongkorn University, Thailand. The cell line was cultivated at 37°C in a 5% (v/v) CO_2_ atmosphere in CM (RPMI-1640 medium with L-Glutamine (HyClone, USA) supplemented with 10% (v/v) fetal bovine serum (HyClone), 100 U/mL penicillin (General Drugs House Co. Ltd., Bangkok, Thailand), 0.4 mg/mL streptomycin (M & H Manufacturing Co. Ltd, Samut Prakan, Thailand) and 1% (w/v) sodium pyruvate (HyClone))

### Drug Assay Procedures

The known diterpene coumarin, clausmarin A [14–16], was the major compound isolated from the leave extract of *C*. *harmandiana* (Pierre) (Yingyongnarongkul B, to be published elsewhere) was used throughout the experiments.

The yeast-based drug assay in liquid culture was performed as previously described [17]. The YNS17 assay cells were inoculated into YPD broth containing various concentrations of clausmarin A at a cell density of ~5 x 10^6^ cells/mL. All assay medium contained a final concentration of 0.5% (v/v) DMSO (the solvent for clausmarin A). Special care was used to add the poorly water-soluble clausmarin A into the culture medium by gradually diluting the clausmarin A in DMSO solution with pre-warmed medium. The YNS17 cell suspensions were pre-incubated for 30 min before adding CaCl_2_ to a final concentration of 100 mM. The cells were then cultivated at 30°C with shaking. The cell density was monitored using a haemacytometer.

### Flow Cytometric Analysis and Nuclear Staining

Progression through the yeast cell cycle was followed using flow cytometric analysis as previously described [18] using a FACS Calibur (Becton Dickinson, Franklin Lakes, NJ) flow cytometer. The yeast cell nuclei were stained with 5 μM Hoechst 33342 (Sigma, St. Louis, MO).

### Reverse Transcription-Polymerase Chain Reaction (RT-PCR)

The expression of IL-2 mRNA was assessed by two-stage semi-quantitative reverse transcription-polymerase chain reaction (sqRT-PCR). The Jurkat T-cell line was inoculated into CM at a density of 0.5 x 10^6^ cells/mL. The cells were treated with various concentrations of clausmarin A for 30 min, and then stimulated with 25 ng/mL of phorbol 12-myristate 13-acetate (PMA) (Merck, Darmstadt, Germany) and 1 μg/mL of ionomycin (Io) (Merck, Darmstadt, Germany) (PMA/Io) at 37°C in 5% (v/v) CO_2_ for 24 h. Total RNA was extracted using the Trizol reagent (Invitrogen, Carlsbad, CA) according to the manufacturer’s protocol. In brief, the cell suspension (1 mL) was harvested by centrifugation at 8000 x g for 5 min, and the cell pellet was suspended in 1 mL trizol reagent. The DNA and protein were separated by chloroform-phase separation and the RNA in the aqueous phase was precipitated with isopropanol and then suspended in diethylpyrocarbonate (DEPC)-treated water. In the first stage, cDNA was synthesized from the obtained total RNA using random hexamer primers (Fermentas, Lithuania) and reverse transcriptase (Thermoscientific, Lithuania). In the second stage gene-specific sqPCR was performed with Taq polymerase using the gene-specific primers for a 380 bp fragment of β-actin (sense, 5’- ACCAACTGGGACGACATGGAGAA-3’ and antisense 5’-GTGGTGGTGAAGCTGTAGCC-3’ [19]), and for a 458 bp fragment of IL-2 (5’-ATGTACAGGATGCAACTCCTGTCTT-3’ and antisense 5’-GTTAGTGTTGAGATG ATGCTTTGAC-3’ [20]). The PCR reaction comprised an initial denaturation step at 94°C for 5 min, followed by 30 cycles of 94°C for 1 min, 55°C for 1 min, 72°C for 1 min. Extension were continued at 72°C for 10 min. The PCR products were separated by 2% (w/v) agarose gel electrophoresis, and the gels were stained with 10 μg/ml Ethidium Bromide for 5–10 min and viewed by UV trans-illumination.

### Assay for IL-2

Jurkat cells were treated as described in the RT-PCR section and then the IL-2 levels in the obtained culture supernatants were measured using the human IL-2 ELISA Ready-SET-Go! kit (eBioscience, San Diego, CA) according to the manufacturer’s instructions.

### Western Blotting Analysis

The phosphorylation/dephosphorylation levels of NFAT in the PMA/Io co-stimulated cells were determined as follows. Jurkat cells were treated with various concentrations of clausmarin A (0 to 100 μM) or with 100 nM FK506 for 30 min at 37°C in CM and 5% (v/v) CO_2_ and then activated by culturing with 25 ng/mL PMA and 1 μg/mL Io in CM at 37°C and 5% (v/v) CO_2_ for 24 h. The treated cells were then harvested, lysed and the total protein content extracted as previously described [21]. The protein concentration was measured using the bicinchoninic acid protein assay (Pierce, Rockford, IL, USA) according to the manufacturer's instructions. The proteins were separated by 10% SDS-PAGE at a constant voltage of 50 V for 3 h and then transferred onto PVDF membrane (BIO-RAD, USA) by Trans-Blot SD Semi-dry transfer cell (BIO-RAD, USA). For Western blotting, the PVDF membrane was blocked in 5% skim milk for 30 min and then incubated for overnight in the respective primary antibody, rabbit anti-NFAT1 antibody (Cell Signaling, USA) or mouse anti-β-actin (Cell Signaling, USA), at a 1:10000 dilution in 5% skim milk. After washing 5 min twice, 15 min twice in PBST blots were incubated for 1 h in the appropriate secondary antibody, donkey anti-rabbit IgG or sheep anti-mouse IgG, conjugated to horseradish peroxidase (Amersham Biosciences, UK), washed as above and then developed by chemiluminescence (Amersham Biosciences, UK).

### Statistical analysis

The unpaired t-test (two-tailed) was performed to determine statistical significance of treated cells and the control cells (GraphPad Prism 5.0).

## Results

### Clausmarin A Alleviates the Compromised Growth of Ca^2+^-Sensitive Δ*zds1* Strain Yeast in High Calcium Medium

**Fig 1:**
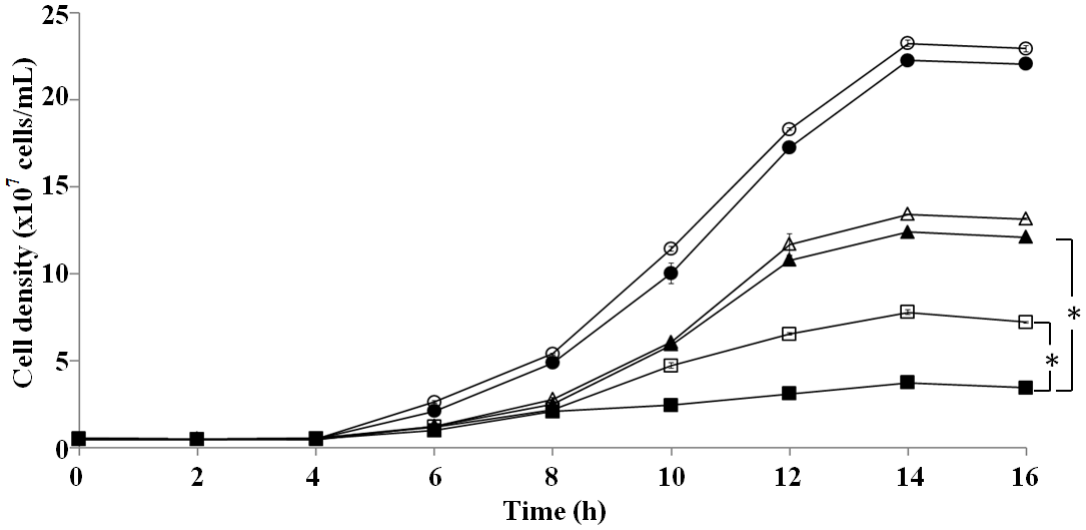
Clausmarin A alleviates the Ca^2+^-dependent growth inhibition of the YNS17 (Δ*zds1*) yeast strain. YNS17 cells (5 x 10^6^ cells/mL) were incubated for 30 min in YPD medium containing the indicated concentration of clausmarin A before the addition of CaCl_2_ to 100 mM final concentration and incubating at 30°C with shaking for the indicated time. Legend: 100 mM CaCl_2_ (■);100 mM CaCl_2_ + 125 μM clausmarin A (□**)**;100 mM CaCl_2_ + 250 μM clausmarin A (▲);100 mM CaCl_2_ + 250 nM FK506 (△); none (○) and 250 μM clausmarin A (●). Data shown are from one trial and are representative of those seen from three independent trials. *Statistically different with *p*-value <0.0001.

Clausmarin A, previously reported terpenoid coumarin from various plant sources [12, 14–16], was rediscovered from the leave extract of *Clausena harmandiana* (Pierre) through a screening of Thai medicinal plants using a yeast-based assay devised for the specific detection of Ca^2+^-signaling inhibitors (Yingyongnarongkul B, to be published elsewhere). The unique feature of this screening procedure is that it facilitates specific detection of inhibitors of the Ca^2+^ signaling pathways by the growth-promoting effect on the compromised growth of yeast cells, suppressing the Ca^2+^-sensitive phenotype of the YNS17 strain yeast on solid YPD medium containing 100 mM CaCl_2_ [11]. Through the screening followed by activity-based purification and structure analysis of an active substance, clausmarin A [12,14–16] was identified as a potential inhibitor of Ca^2+^-signaling in yeast (Yingyongnarongkul B, to be published elsewhere). To further investigate this phenomenon in liquid culture, the growth of YNS17 cells was monitored in YPD medium containing 100 mM CaCl_2_ in the presence of varying concentrations (0 to 250 μM) of clausmarin A (Fig 1). YNS17 cells cultured without CaCl_2_ treatment increased in number from 4 h to a maximal level (stationary phase) at 14–16 h and this was not affected by treatment with 250 μM clausmarin A, suggesting that the drug by itself had no toxic nor growth-promoting effect (Fig 1). Treatment with 100 mM CaCl_2_ almost completely inhibited the growth of the YNS17 cells, due to the activation of the Ca^2+^-signaling pathways by the external CaCl_2_. However, cell growth in the presence of CaCl_2_ was partially restored by 125 and 250 μM clausmarin A treatment in a concentration-dependent manner. The extent of restoration was similar to that obtained with 250 nM FK506, a known potent inhibitor of calcineurin.

### Effects of Clausmarin A on Genetically Manipulated Yeast Strains

**Fig 2:**
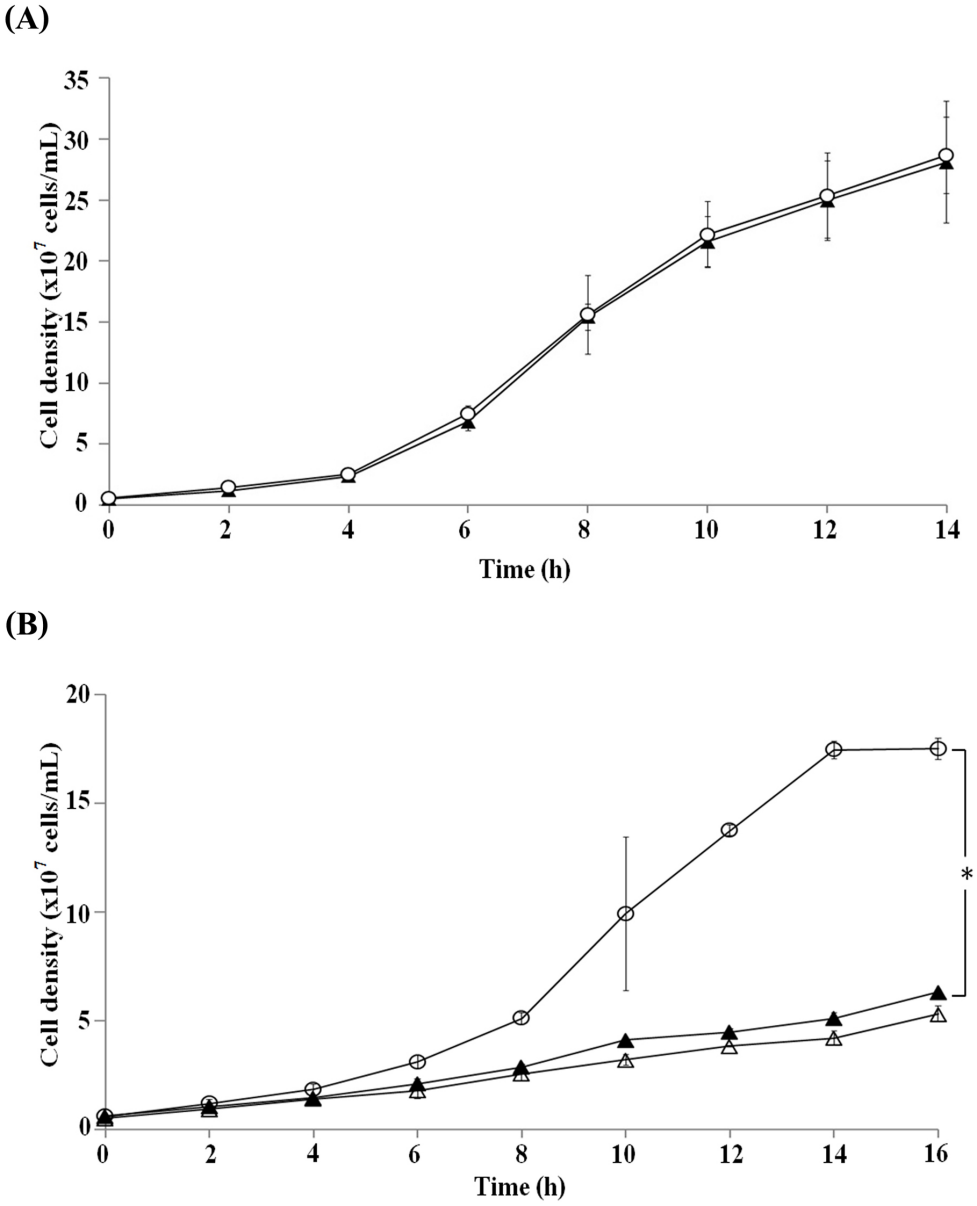
Clausmarin A inhibits the calcineurin but not the Mpk1 pathway. The effect of clausmarin A on the growth of the loss-of-function mutations in the (**A**) calcineurin (Δ*cnb1*) or (**B**) the Mpk1 MAP kinase (Δ*mpk1*) Ca^2+^-signaling pathways. The experimental procedures were similar to that described in the legend to Fig 1, except no CaCl_2_ was added. Legend: no addition (○); 250 μM clausmarin A (▲); 250 nM FK506 (△). Data shown are from one trial and are representative of those seen from three independent trials. *Statistically different with *p*-value <0.0001.

**Fig 3:**
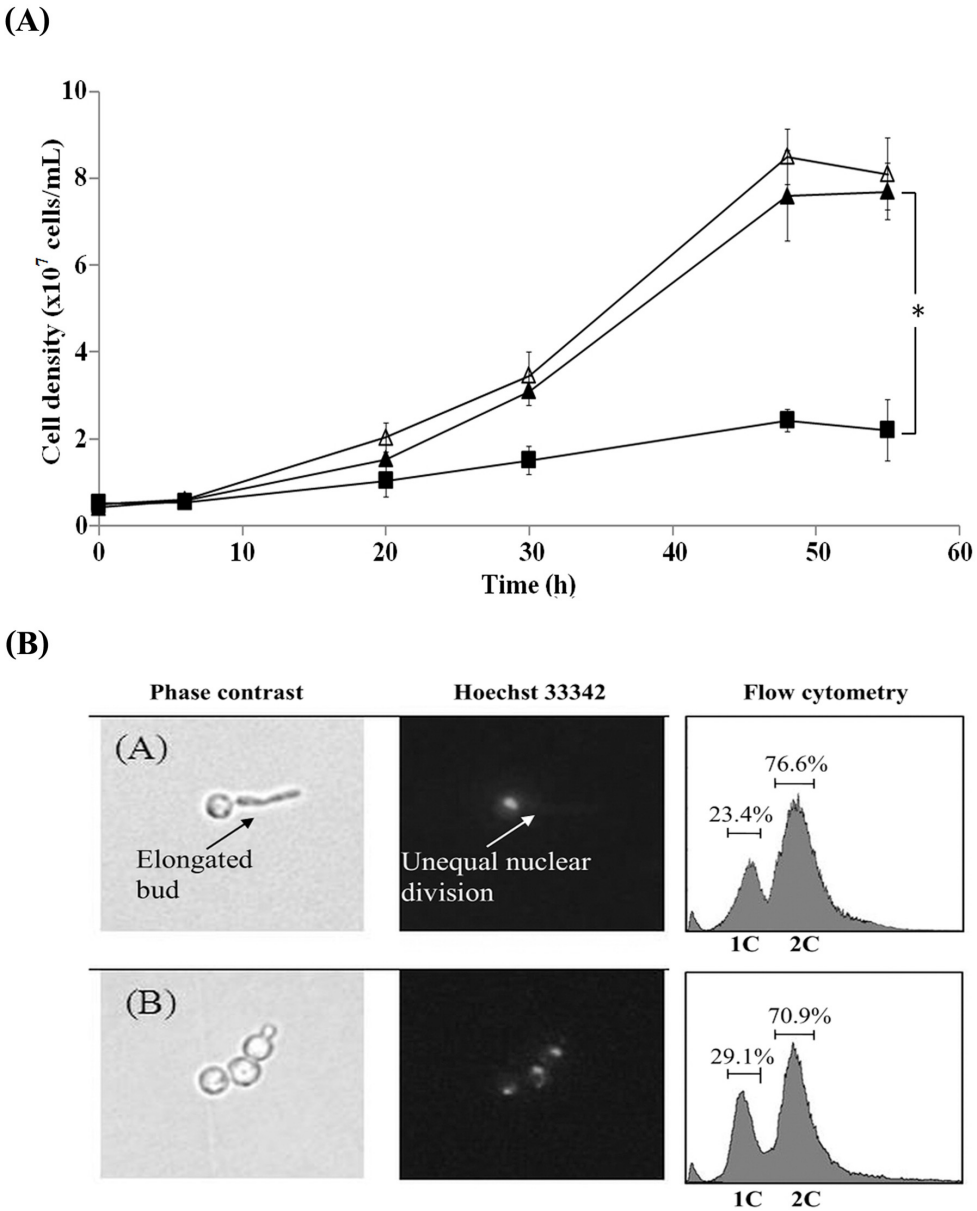
Clausmarin A alleviates the growth inhibition of a constitutively active calcineurin. **(A)** The effect of clausmarin A on the YRC1 yeast strain expressing an inducible constitutively active form of calcineurin. The experimental procedures were similar to those described in the legend to Fig 1, except the YRC1 strain with a chromosomally integrated construct for the galactose-inducible constitutively active form of the calcineurin catalytic subunit (*CMP2*ΔC) and the appropriate medium (SC) without CaCl_2_ addition for induction of *GAL1* promoter was used. Legend: SC medium with no addition (□); 250 μM clausmarin A (▲); 250 nM FK506 (△). The *GAL1* promoter was induced by the addition of 2% (w/v) galactose and incubated at 30°C with shaking for the indicated period of time. *Statistically different with *p*-value <0.001. **(B)** The samples obtained after 12 h of incubation were observed under phase-contrast microscopy (left), fluorescence microscopy of Hoechst 33342-stained cells (middle) or flow cytometric analysis of Hoechst 33342-stained cells (right). Data shown are from one trial and are representative of those seen from three independent trials.

In the yeast Ca^2+^-signaling, the parallel calcineurin- and Mpk1-mediated pathways redundantly regulate some events essential for cell growth and morphogenesis [20]. Thus, a loss-of-function in either branch of the two parallel pathways is still viable (*e*.*g*., Δ*cnb1* single mutant lacking the calcineurin pathway or Δ*mpk1* single mutant lacking the Mpk1 MAP kinase pathway), but the simultaneous loss-of-function in both branches is lethal (*e*.*g*., Δ*cnb1* Δ*mpk1* double mutant). Therefore, to distinguish whether the calcineurin branch or the Mpk1 branch is likely to be inhibited by clausmarin A, the synthetic lethality due to clausmarin A was evaluated on each of the Δ*cnb1* and Δ*mpk1* single mutant yeast strains. The Δ*cnb1* and Δ*mpk1* cells were cultured in YPD medium in the presence of 250 μM clausmarin A or 250 nM FK506 and the cell growth was monitored (Fig 2). The growth of the Δ*cnb1* cells was unaffected by the inclusion of clausmarin A or FK506, indicating that the Mpk1 pathway is not likely to be inhibited by these drugs (Fig 2A). In contrast, the growth of the Δ*mpk1*cells was severely inhibited (> 72%) by the inclusion of clausmarin A (250 μM) or FK506 (250 nM) to a similar extent (Fig 2B), implying that clausmarin A, similar to FK506, inhibited the calcineurin but not the Mpk1 pathway. If the target of clausmarin A is in the calcineurin branch, then it could be predicted that the drug will alleviate the deleterious effects caused by the expression of a constitutively active form of calcineurin, which otherwise leads to similar changes to that observed with the YNS17 cells treated with 100 mM CaCl_2_ [22]. The YRC1 strain (Δ*zds1* + [*GAL1p-CMP*2ΔC]), which over-expresses a constitutively active form of the catalytic subunit of calcineurin (Cmp2ΔC) upon galactose induction, was first treated with 250 μM clausmarin A (or 250 nM FK506) for 30 min and then 2% (w/v) galactose was added to induce the expression of the calcineurin gene (Fig 3A). As expected, a severe growth retardation (> 78%) resulted from the forced expression of the constitutively active form of calcineurin, but this was alleviated by the treatment with either 250 μM clausmarin A or 250 nM FK506 to a broadly similar extent, suggesting that calcineurin or a functionally closely related molecule is the target of clausmarin A. Hyper-activation of Ca^2+^ signals in Δ*zds1* cells (by externally supplemented CaCl_2_ or by *CMP2*ΔC overexpression) not only causes growth retardation, but also induces polarized bud growth and cell-cycle arrest in the G2 phase in affected cells [18]. Thus, whether clausmarin A could alleviate the altered morphology and the cell-cycle arrest caused by *CMP2*ΔC overexpression was also evaluated (Fig 3B). The abnormal budding morphology (polarized bud growth), as observed by a phase contrast microscopy, and the unequal nuclear division, as visualized in Hoechst 3342-stained cells using fluorescence microscopy, 6 h after *CMP2*ΔC induction, were both alleviated by treatment with 250 μM clausmarin A. The Ca^2+^-induced G2 arrest, as measured by FACS analysis, showed an increase in the 1C peak (from 23.4% to 29.1%) and a decrease in the 2C peak (from 76.6% to 70.9%), indicating that the G2 cell-cycle arrest was also alleviated (Fig 3B). These results demonstrated that each of the abnormalities caused by activated calcineurin was suppressed by clausmarin A, supporting the notion that the drug inhibited the calcineurin pathway in the yeast. Similar results were obtained with 250 nM FK506 (data not shown; [18], [23]).

### Inhibition of IL-2 Production and IL-2 Gene Transcription by Clausmarin A in the Human Jurkat T-Cell Line

Based on the finding that clausmarin A is a likely inhibitor of the evolutionary conserved calcineurin-mediated pathway in yeast, this finding was then evaluated in mammalian cell system using Jurkat human T leukemia cells. Calcineurin plays a key regulatory role in the production of IL-2 during T-cell activation, including the activation of Jurkat cells with PMA/Io. The toxicity of clausmarin A to Jurkat cells was evaluated first to select non-toxic concentrations. Jurkat cells were treated for 24 h in CM containing clausmarin A at various concentrations (0–100 μM) and the cell viability was measured by the MTT viability assay [24]. Clausmarin A did not inhibit the Jurkat cell growth at concentrations up to 10 μM, but at 100 μM a weak toxicity (~20% loss of viability) was noted.

**Fig 4:**
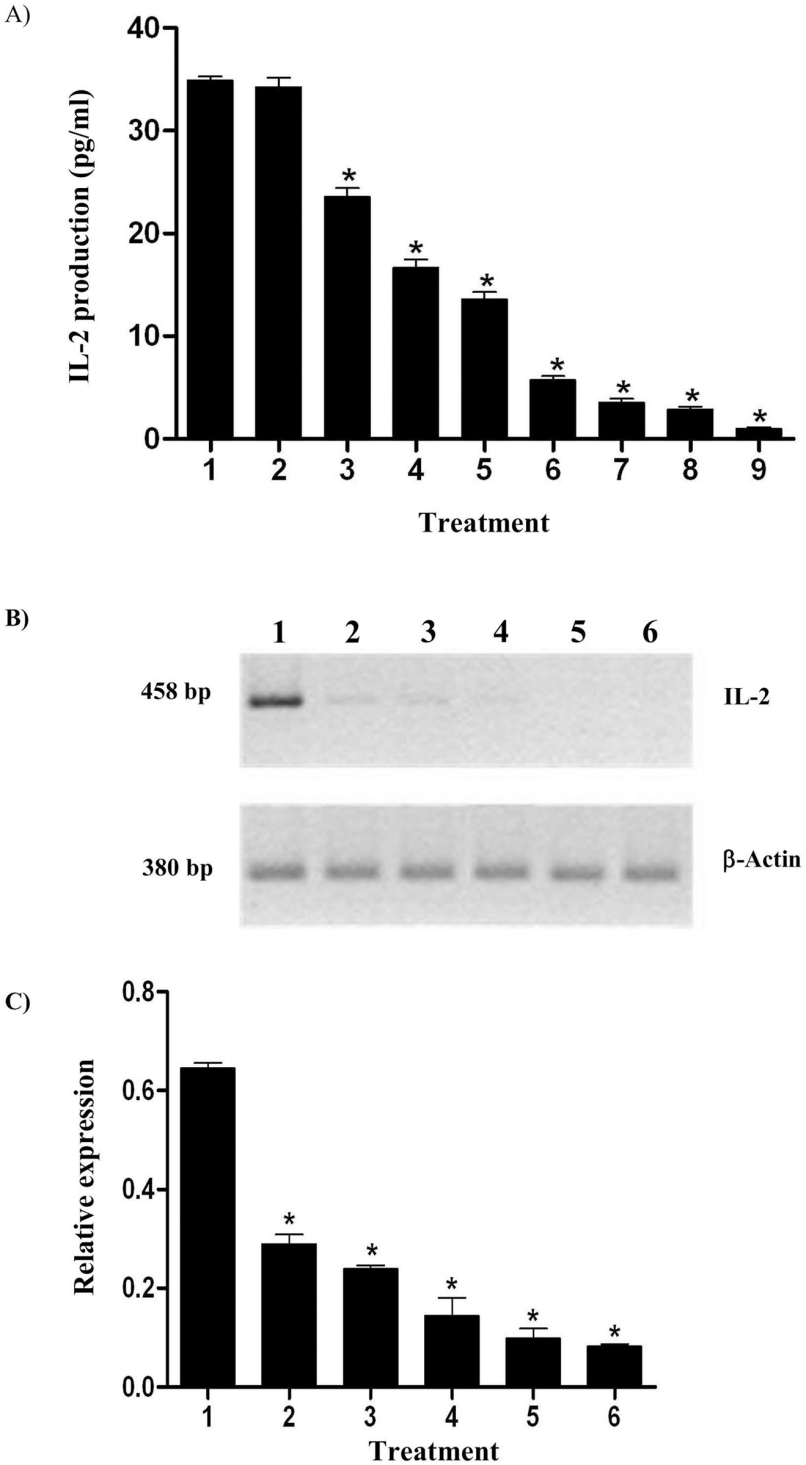
Clausmarin A inhibits IL-2 and IL-2 mRNA production in Jurkat cells. **(A)** Jurkat cells were treated with various concentrations of clausmarin A or with 100 nM FK506 for 30 min, then stimulated with 25 ng/mL PMA and 1 μg/mL Io at 37°C for 24 h. The treatments were: (1) none, (2) 0.5% (v/v) DMSO, (3–7) clausmarin A at (3) 0.25 μM, (4) 1 μM, (5) 2.5 μM, (6) 5 μM and (7) 25 μM, (8) 100 nM FK506 and (9) unstimulated (no PMA/Io treatment). (**B)** Effect of clausmarin A on IL-2 mRNA expression. Jurkat cells were treated with various concentrations of clausmarin A or with 100 nM FK506 for 30 min prior to stimulation with PMA/Io as described in **(A**). The various treatments were: (1) none, (2–4) clausmarin A at (2) 5 μM, (3) 25 μM and (4) 50 μM, (5) 100 nM FK506 and (6) unstimulated (no PMA/Io treatment). RNA was extracted and subjected to two-stage gene specific sqRT-PCR. (**C**) Corrected IL-2 expression levels relative to that of β-actin. Data are shown as the mean ± 1 SD, derived from three replicated. Means that are significantly different from the untreated cells at *p* ≤ 0.001 are indicated by *.

During T-cell activation, the nuclear translocation of NFAT from the cytoplasm is dependent on its dephosphorylation by calcineurin. The classical calcineurin inhibitors cyclosporin A and FK506 inhibit this calcineurin phosphatase activity and thus prevent the nuclear translocation of NFAT. Since NFAT is a crucial transcriptional activator in T cell activation, its inhibition suppresses the T-cell immune responses [25]. The effect of clausmarin A on the IL-2 production in Jurkat cells was evaluated by treating the cells with various concentrations of clausmarin A (0.25 to 25 μM) prior to stimulation with PMA/Io for 24 h at 37°C in CM with a 5% (v/v) CO_2_ atmosphere. The supernatant was collected and assayed for IL-2 content by indirect ELISA. As a control Jurkat cells were treated with 100 nM FK506, which strongly inhibited IL-2 production. Clausmarin A dose-dependently inhibited IL-2 production (Fig 4A).

With respect to the effect of clausmarin A on the expression levels of IL-2 mRNA, as determined by two-stage sqRT-PCR (Fig 4), in the untreated control cells the 458 bp PCR product was clearly detected, but the transcript level was diminished by clausmarin A in a dose-dependent manner. The mRNA band was barely detectable in the cells treated with 100 nM FK 506. When these results were quantified relative to the β-actin transcript levels (Fig 4C) they clearly demonstrated that clausmarin A, similar to FK506, inhibited IL-2 gene expression at the transcriptional level.

### Inhibition of NFAT Dephosphorylation by Clausmarin A

**Fig 5:**
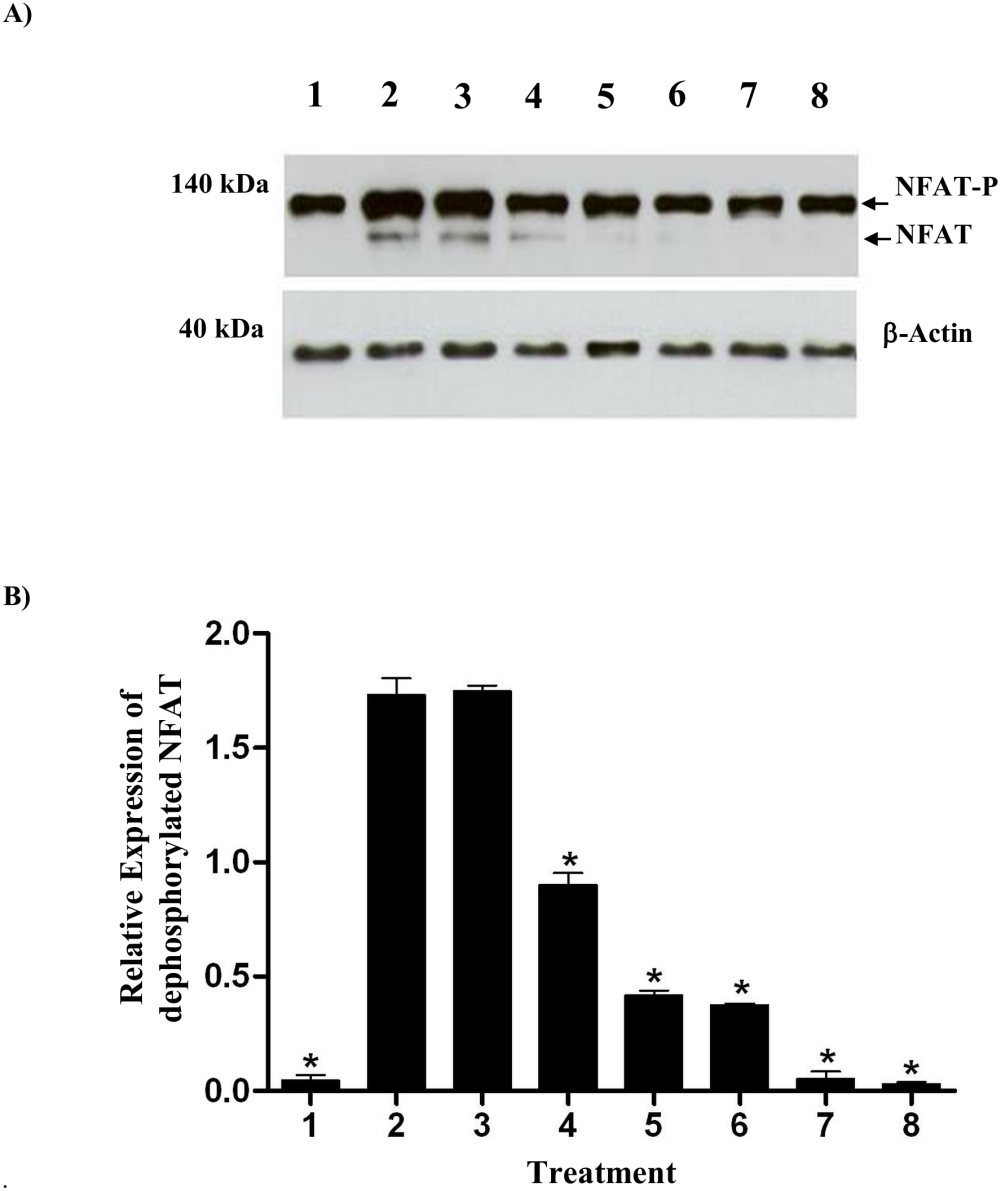
Clausmarin A inhibits NFAT dephosphorylation in Jurkat cells. Jurkat cells were treated with various concentrations of clausmarin A or FK506 for 30 min at 37°C and then activated with 25 ng/mL PMA and 1 μg/mL Io at 37°C for 24 h. The treatments were: (1) unstimulated, (2–8) PMA/Io stimulated following treatment with (2) none, (3–7) clausmarin A at (3) 12.5 μM, (4) 25 μM, (5) 50 μM, (6) 75 μM and (7) 100 μM, and (8) 100 nM FK506. **(A**) Western blot showing the dephosphorylated and phosphorylated forms of NFAT and that of the β-actin loading control. Data are representative of three replicate experiments **(B)**. The relative amount of dephosphorylated NFAT to the β-actin loading control. Data are shown as the mean ± 1 SD, derived from three replicated. Means that are significantly different from the untreated cells at *p* ≤ 0.001 are indicated by *.

Whether the inhibition of the IL-2 gene expression by clausmarin A is due to the inhibition of NFAT dephosphorylation was evaluated by examining the phosphorylation state of NFAT by Western blot analysis. Jurkat T cells were treated with clausmarin A (12.5 to 100 μM) for 30 min prior to being stimulated with PMA/Io in CM for 24 h at 37°C and 5% (v/v) CO_2_. Cell extracts were prepared and subjected to Western blot analysis using anti-NFAT1 and anti-actin primary antibodies to detect NFAT and β-actin (Fig 5). In the unstimulated cells, NFAT was found exclusively in the phosphorylated form, reflecting that calcineurin is inactive under resting conditions. In contrast, in the PMA/Io stimulated cells in the absence of clausmarin A the dephosphorylated form of NFAT was detected as well as the phosphorylated form. The presence of 100 nM FK506 strongly inhibited the dephosphorylation of NFAT. Clausmarin A showed a dose-dependent inhibition of NFAT dephosphorylation. Thus, clausmarin A, similar to FK506, inhibited the calcineurin-dependent dephosphorylation step of NFAT, a critical step in IL-2 expression. A similar pattern of the inhibition of NFAT dephosphorylation has been reported previously for other pyrazole compounds [3].

## Discussion

Using the Δ*zds1* strain yeast-based screening assay, clausmarin A, a terpenoid coumarin, exhibited an apparent Ca^2+^-signaling inhibitory activity (Yingyongnarongkul B, to be published elsewhere). This compound had been reported as a component in many plants in genus *Clausena e*.*g*. from leaves of *Clausena heptaphylla* [15] and *C*. *pentaphylla* [14, 16]. Clausmarin A was reported to induce a spasmolytic activity on isolated guinea-pig ileum preparations [12], but its function at the molecular level was unknown.

In this study, the calcineurin-mediated pathway in the Ca^2+^-mediated cell-cycle regulation in yeast was identified as the potential target of clausmarin A. Given that calcineurin is highly conserved from yeast to human, the effect of clausmarin A on human cells was evaluated using the human Jurkat leukemic T-cell line, in which the function of calcineurin can be measured by the activation of the IL-2 gene expression in the activated cells, including after PMA/Io stimulation. Indeed, clausmarin A inhibited in a dose-dependent manner the IL-2 production and mRNA level in PMA/Io-stimulated Jurkat cells. Moreover, the dephosphorylation of phosphorylated NFAT, a critical step in the activation of IL-2 gene expression, was inhibited by clausmarin A. Overall, the data obtained in the yeast and human Jurkat cell systems indicated that clausmarin A is an inhibitor of the calcineurin-mediated pathway. In fact, the behaviors of clausmarin A and FK506 in the yeast and Jurkat cell systems were broadly similar in all tested assays.

The biologically effective concentrations of clausmarin A in yeast were almost three orders of magnitude higher than those in Jurkat cells (compare Figs 1 and 4 for yeast and Jurkat cells, respectively). It is known that yeast is highly resistant to drugs in general compared to mammalian cells. Yeast cells are surrounded by thick cell wall and the yeast plasma membranes contain ergosterol instead of cholesterol of the mammalian counterparts. The unique features of the yeast cell surface are thought to contribute to the high degree of drug resistance serving as a drug barrier [26]. In addition, it has been known that yeast can acquire resistance to various unrelated drugs. Multidrug resistance is caused by the increased expression of various genes that encode nonspecific drug-efflux pumps that belong to the ABC family of transporter proteins [27]. Presumably due to these and some more mechanisms, much higher doses of clausmarin A are needed in yeast compared to the Jurkat cells.

Based on the *in vivo* effects of clausmarin A in yeast and Jurkat cells, the direct *in vitro* inhibition of calcineurin phosphatase activity by clausmarin A was assessed using a calcineurin assay kit (Calbiochem) that employs human recombinant calcineurin. However, ~60% of the calcineurin activity remained in the presence of 250 μM clausmarin A, a concentration that is effective in the yeast *in vivo* system, in contrast to the complete inhibition of calcineurin by 150 nM FK506 (S1 Fig). The low level of direct *in vitro* inhibition of calcineurin by clausmarin A could be due to the lack of an as yet unidentified immunophilin-like protein in the assay system, which may be required for the inhibition of calcineurin by clausmarin A. An approach to identify the protein(s) that bind to clausmarin A is currently under way.

The concentrations of clausmarin A for eliciting biological activity in yeast and Jurkat T cells were much higher than those of FK506, with a difference almost three orders of magnitude in each of the cell systems (*e*.*g*., Fig 1 for yeast and Fig 4 for Jurkat cells). An attempt to develop clausmarin A derivatives that show higher specific activity is in progress in our laboratory.

Although the toxicity of clausmarin A has not been formally addressed, it showed no acute cytotoxicity to Jurkat T cells up to dosage of 100 μM. This emphasized the potential for further development of clausmarin A as an immunosuppressive drug, albeit subject to proper toxicity evaluation.

Our studies clearly demonstrated that the yeast-based assay can offer powerful means for the discovery of the compounds of medicinal interest and the elucidation of drug-action mechanisms, owing to the power of molecular genetic techniques available in this organism. The beneficial features of using yeast also derive from the fact that many small molecule inhibitors act on common target molecules in an evolutionally conserved manner throughout eukaryotes. As shown in this study for the Ca^2+^-signaling pathways, investigating the related pathways in yeast and human cells in parallel could provide an effective methodology for discovering and studying the action mechanism of medicinally interesting small-molecule compounds.

## Supporting Information

S1 Fig No significant effect of clausmarin A on calcineurin activity *in vitro* at the physiologically effective drug concentrations. Treatments were as follows: 1) H_2_O, 2) 0.5% DMSO, 3) 150 nM FK506, 4–7) Clausmarin A at 500 μM (4), 250 μM (5), 125 μM (6) and 62.5 μM (7). The *in vitro* calcineurin activity assay using the calcineurin assay kit (Calbiochem., Darmstadt, Germany) which employs recombinant human calcineurin according to the manufacturer’s instructions was tested and the free Pi release was detected. *Statistically different with *p*-value *p* ≤ 0.001. Although the physiological effect of clausmarin A in Jurkat cells was observed at a concentration range of 0.25 to 25 μM (Fig 4), a dose-dependent inhibitory effect on the calcineurin activity *in vitro* was detected only at much higher concentrations as above 62.5 μM, suggesting that clausmarin A does not inhibit calcineurin *in vitro* at the physiologically relevant clausmarin A concentrations (see Discussion).
(TIF)
