# Supplementary figures and images for: Clausmarin A, Potential Immunosuppressant Revealed by Yeast-Based Assay and Interleukin-2 Production Assay in Jurkat T Cells
Source: PLoS One. 2015 Aug 27;10(8):e0136804. doi: 10.1371/journal.pone.0136804 (PMC4552291; doi:10.1371/journal.pone.0136804)

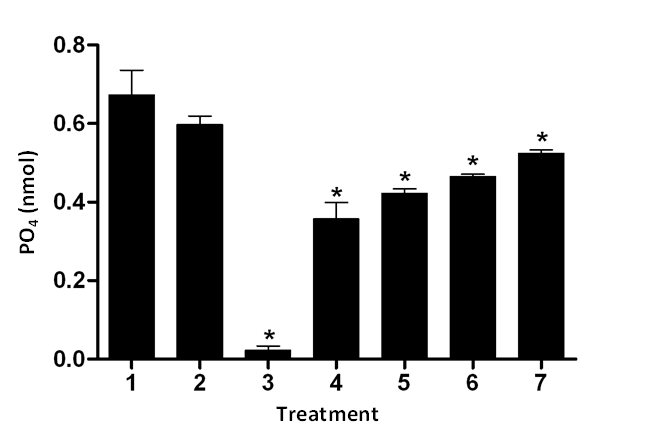

Supplement: S1 Fig — Treatments were as follows: 1) H2O, 2) 0.5% DMSO, 3) 150 nM FK506, 4–7) Clausmarin A at 500 μM (4), 250 μM (5), 125 μM (6) and 62.5 μM (7). The in vitro calcineurin activity assay using the calcineurin assay kit (Calbiochem., Darmstadt, Germany) which employs recombinant human calcineurin according to the manufacturer’s instructions was tested and the free Pi release was detected. *Statistically different with p-value p ≤ 0.001. Although the physiological effect of clausmarin A in Jurkat cells was observed at a concentration range of 0.25 to 25 μM (Fig 4), a dose-dependent inhibitory effect on the calcineurin activity in vitro was detected only at much higher concentrations as above 62.5 μM, suggesting that clausmarin A does not inhibit calcineurin in vitro at the physiologically relevant clausmarin A concentrations (see Discussion). (TIF) [file pone.0136804.s001.tif]
